# Advancing a new model of collaborative practice: a decade of Whole Health interprofessional education across Veterans Health Administration

**DOI:** 10.1186/s12909-024-05945-7

**Published:** 2024-09-10

**Authors:** Theresa H. Liao, J. Adam Rindfleisch, Kelly Peyton Howard, Marc Castellani, Sara Grimsgaard Noyes

**Affiliations:** 1 Office of Patient Centered Care and Cultural Transformation, Veterans Health Administration, Washington, DC USA; 2 Department of Family Medicine and Community Health, University of Wisconsin, Madison, WI USA; 3 US Army Training and Doctrine Command, Holistic Health and Fitness Directorate, Richmond, Virginia USA; 4 Section of General Medicine, Portland VA, Portland, OR USA

**Keywords:** Interprofessional education, Collaborative practice, Whole health, Interprofessional care, Transdisciplinary care, Interdisciplinary care, Transformative education, Integrative medicine, Integrative health, Health care transformation

## Abstract

Large-scale implementation of interprofessional education across the United States Veterans Health Administration has supported advancement of a new model of collaborative practice, the Whole Health System, centering on the patient and what matters most to them. Other health care systems can consider similar educational efforts for health care transformation.

## Introduction: Whole Health collaborative practice

In 1996, the United States (US) Institute of Medicine recommended that primary care teams follow an interdisciplinary model wherever feasible to optimize responsiveness of care [1]. Since then, others have called for more advanced models of interprofessional care, including transdisciplinary care, characterized by even greater collaboration and ongoing cross-disciplinary education, with explicit inclusion of patient and family at all stages as team members, as well as transcending disciplinary boundaries and focusing on real-world problems [2, 3]. In 2010, the World Health Organization (WHO) released its *Framework for Action on Interprofessional Education and Collaborative Practice* in response to fragmented and under-resourced health systems worldwide that were unable to meet the health needs of their communities [4]. Interprofessional education occurs when “students from two or more professions learn about, from and with each other to enable effective collaboration and improve health outcomes.” This results in a “collaborative practice-ready” health workforce, where health workers optimize each other’s skills to reduce fragmentation and strengthen health systems, and work together with patients, their families, and communities, ultimately leading to improved health outcomes. The report highlighted evidence related to effective interprofessional education and care and importantly, called for integrated health and education policies [4]. 

**Fig. 1:**
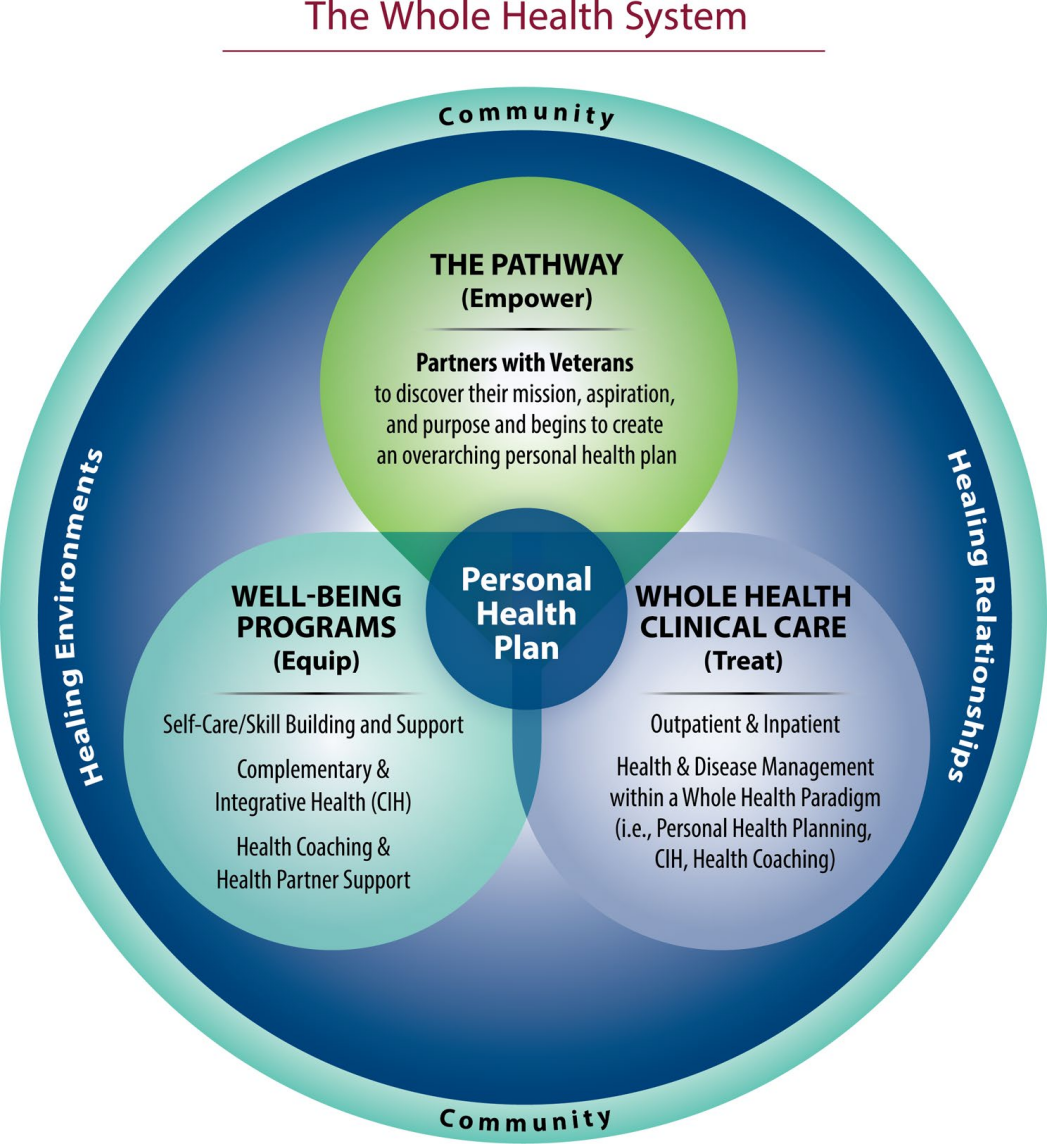
The Whole Health System. The Whole Health System has three distinct parts- Whole Health Clinical Care, Well-Being Programs, and The Pathway, with the Veteran’s personal health plan connecting all parts of care. New interprofessional team members help support Veterans throughout the entire system, with the explicit alignment of all care with what matters most to the Veteran

**Fig. 2:**
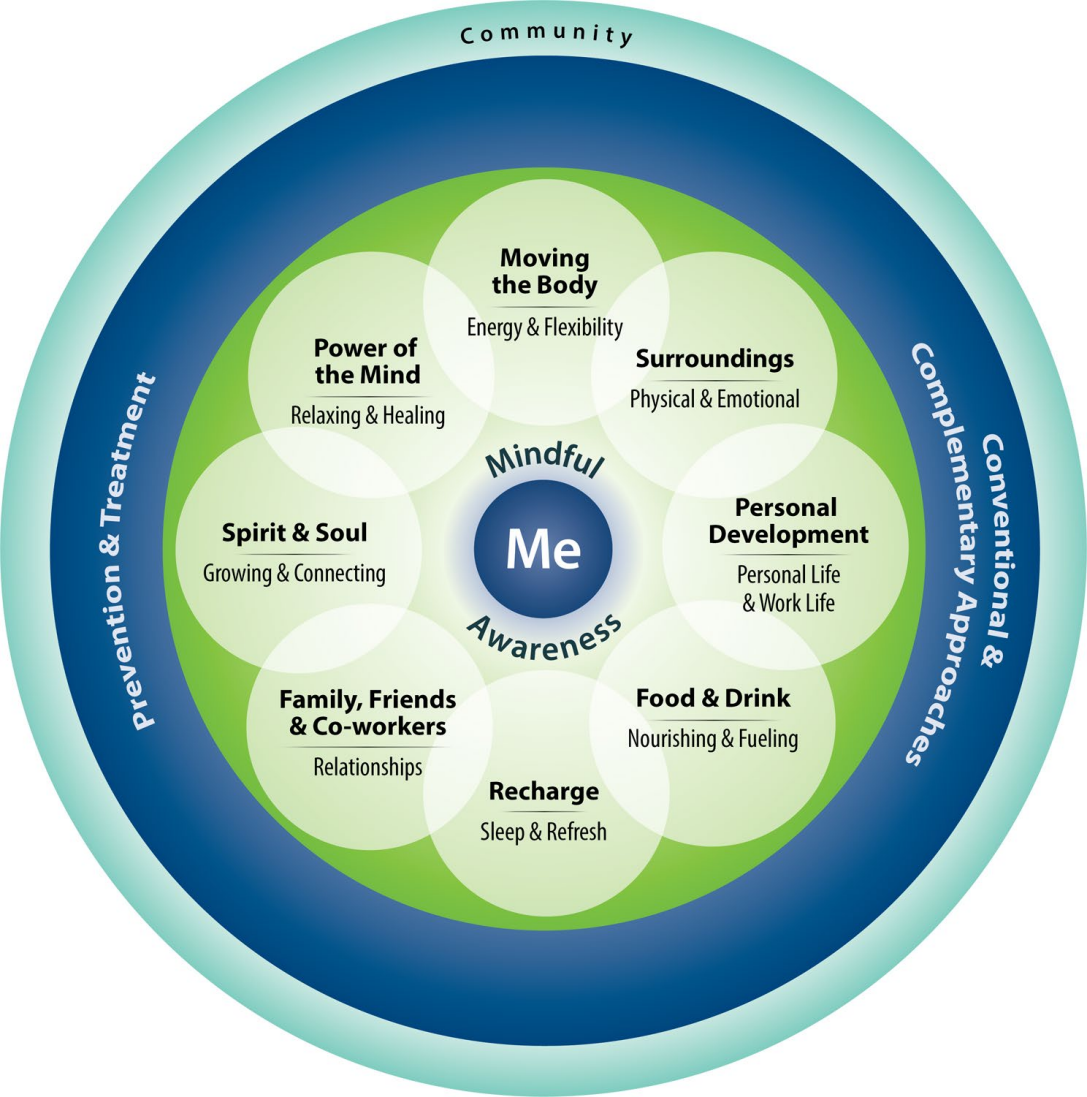
The Circle of Health and Well-Being. The Circle of Health and Well-Being depicts components of proactive health and well-being and is used with Veterans and employees alike. It illustrates the significant contributions individuals can make to their own health and well-being, activated by intentional reflection on what really matters to them, and aided by their care teams and communities

**Fig. 3:**
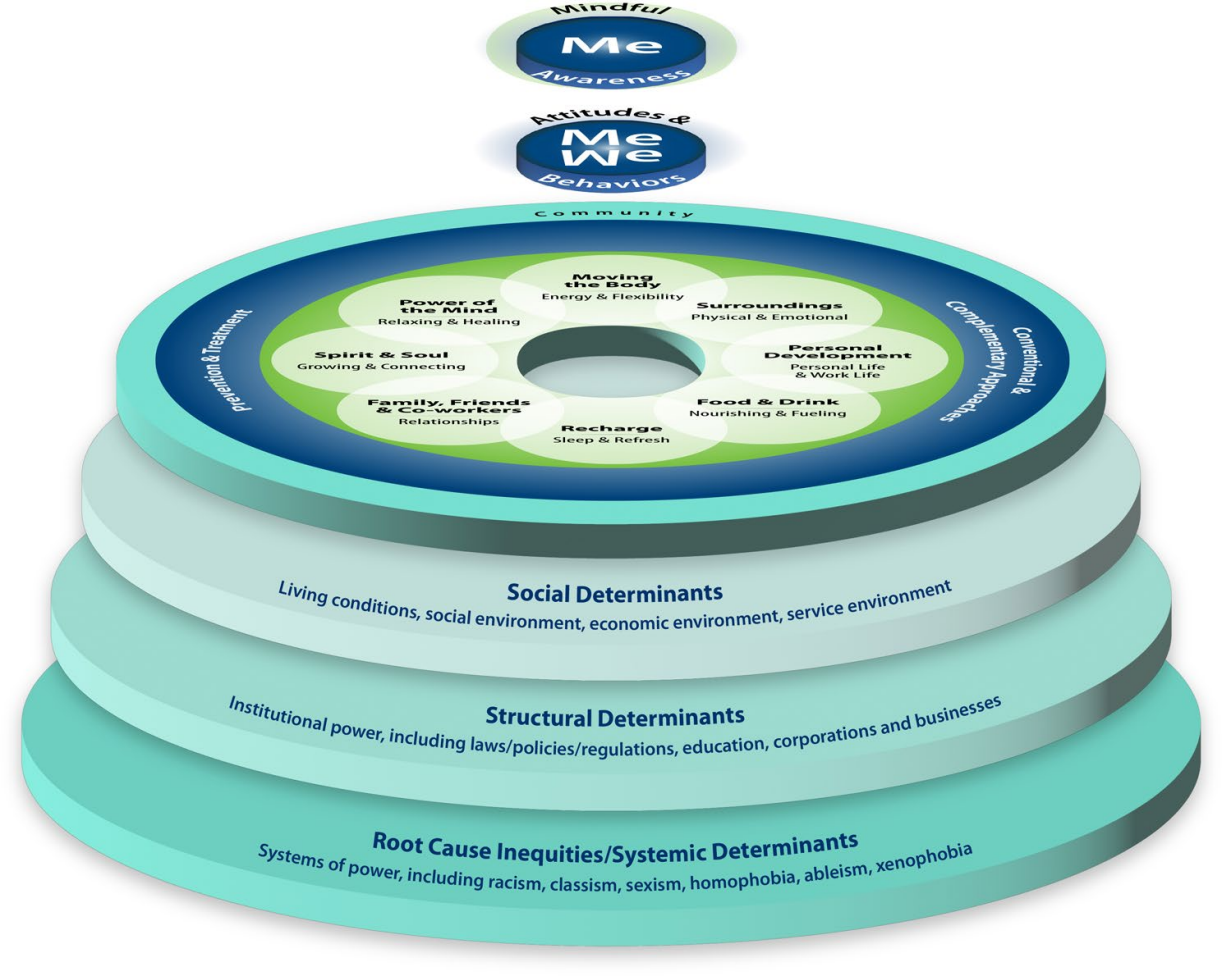
Whole Health for All. The Whole Health for All graphic adds more depth to the Circle of Health and Well-Being by encouraging assessment of the interplay of individual choice, societal influence and root causes that drive health outcomes, supporting health care interventions that eliminate health inequities

The US Veterans Health Administration (VHA) serves people “who served in the active military, naval, or air service” and were not dishonorably discharged [5]. There are over 16 million Veterans in the US, and over 9 million of them are enrolled with VHA, which includes 172 medical centers and over 1,130 outpatient sites [6]. Veterans are diverse in terms of demographics such as race, gender, socioeconomic status, age, and where in the US they live [7, 8]. In 2011, VHA, both the largest integrated health care system and the largest site for health professions training in the US, embarked on an ambitious journey to transform its health care system into one that supports a Whole Health approach, defined as person-centered, whole-person care that empowers and equips Veterans to take charge of their own health and well-being to live their fullest lives (Fig. 1) [6, 9, 10]. In order to support this approach to care, VHA developed additional infrastructure, implementing a Whole Health System of care (Fig. 1) that includes three parts: (1) Whole Health Clinical Care in which clinicians provide excellent whole person care that aligns with what really matters most to each Veteran; (2) Well-Being Programs, which provide access to diverse resources for learning skills in supporting one’s own health and well-being (mindfulness, yoga, nutrition, etc.) and offer evidence-based complementary and integrative health (CIH) approaches; and (3) The Pathway, in which Veterans support fellow Veterans in a peer relationship exploring what really matters to them, defined by their Mission, Aspiration and/or Purpose (MAP) [11]. A Veteran’s MAP is central to aligning the three parts of the Whole Health System to create a Veteran’s personal health plan. Creation of the plan is commonly supported using the Circle of Health and Well-Being (Fig. 2). VHA’s Whole Health approach also incorporates the significant contributions of social, structural, and systemic determinants of health for its diverse population of Veterans (Fig. 3). Interprofessional care, including from team members not previously considered central in most health care systems, is foundational in the Whole Health System.

## Expanding the interprofessional care team

VHA has long utilized a model of interprofessional care for its complex population. A single Veteran may be cared for by team members from multiple disciplines and backgrounds, such as a Veteran living with depression, chronic pain, and advanced diabetes whose care team includes a primary care provider, nurse care manager, clinical pharmacist, dietician, psychiatrist, psychologist, social worker, ophthalmologist and nephrologist, as well as various students, interns, residents, fellows, and other learners. Adopting the Whole Health approach to care in the VHA has advanced interprofessional care to include support from all three parts of the Whole Health system (i.e.: Whole Health Clinical Care, Well-Being Programs, and The Pathway, Fig. 1), working seamlessly to enhance all aspects of health and well-being through collaborative practice aligned with what really matters to each Veteran. For the Veteran mentioned above living with depression, chronic pain and advanced diabetes, the care team in the Whole Health System may now also include a peer Whole Health Partner, a fellow Veteran who has been trained in Whole Health principles, to explore what really matters most to the Veteran and the reasons why their health is important to them. For example, discussion with the Whole Health Partner may reveal that what really matters to this Veteran with multiple complex health issues is their family, and being able to continue to work because they are the sole source of income for their family. This type of exploration may be valuable in strengthening a patient’s engagement in their own health care [12]. 

Additionally, this information is shared by the Whole Health Partner with the care team, who may then refer the Veteran to additional team members to provide other support, such as a Mindfulness-Based Stress Reduction teacher to manage the chronic negative emotions of trying to manage one’s blood sugars on an ongoing basis (diabetes distress), and a tai chi instructor to support moving the body for chronic pain as well as diabetes management (both Mindfulness-Based Stress Reduction and Tai Chi are now recommended by a 2023 diabetes clinical practice guideline) [13]. Referral to a health and wellness coach to find clarity around values, meaning, and purpose, and to create sustainable health goals with personalized support, action planning and accountability may also be helpful, even as the Veteran continues to work with all of their previous health care team members [14]. Explicitly at the center of all of this care is what really matters to the Veteran. The Whole Health model of care intentionally and robustly centers important forces that have always had critical roles in health and well-being, but typically have not been given equal power in the health care system: Veterans themselves, their families and loved ones, and their communities. How diverse team members can work together in coordinated, effective, patient-centered practice to promote the health and well-being of patients in a way that is aligned with what really matters to the patient may not be clear for the many team members involved, especially given the addition of novel roles introduced as part of the Whole Health System. Interprofessional education about a Whole Health approach has therefore been developed and spread across VHA to help with this gap in knowledge and skills related to collaborative practice in support of Whole Health transformation.

To elaborate, key new interprofessional team members in the Whole Health System include the following:

### Complementary and integrative health (CIH) practitioners

CIH practitioners include acupuncturists; massage therapists; practitioners who offer clinical hypnosis, biofeedback, and guided imagery; and instructors of yoga, tai chi, and mindfulness. These services (which may be offered individually or in groups) have been part of the standard benefits package for all Veterans across all VHA facilities since 2017 [15]. In addition, chiropractic care has been widely available to Veterans since 2004 [16]. A growing body of literature describes the importance of these disciplines which have historically not been well integrated within conventional models of care within the US. Mindfulness, yoga, acupuncture, massage and spinal manipulation are now part of evidence-based guidelines for chronic low back pain, the leading cause of disability globally, and evidence supports CIH use for a variety of conditions [17–23]. While a substantial number of patients use CIH, many US clinicians lack knowledge and experience in discussing these approaches with patients, and conversely, patients who use CIH often do not disclose CIH use to their clinical care team members [24–26]. Effective interprofessional care has the potential to address this concerning pattern and support more person-centered, evidence-based care with improved clinical outcomes.

### Health and wellness coaches

The leading causes of death and disability in the US and globally can be traced back to a few foundational health behaviors, and health promotion and disease prevention have been important long-standing goals in population health [27–29]. Health and wellness coaching is a newer profession, intended to support people with changing their health behaviors and realizing their personal health goals; it is often embedded within health care systems [30]. As defined by the National Board of Health and Wellness Coaching, “Health & wellness coaches support clients in activating internal strengths and external resources to make sustainable and healthy lifestyle behavior changes. Health coaches use a client-centered approach wherein clients decide their goals, engage in self-discovery or active learning processes, and self-monitor behaviors to increase accountability, all within the context of an interpersonal relationship with a health coach.” [31] While further research is needed, health coaching has been linked to improved self-care practices and has shown promise contributing to clinical improvements for many chronic conditions [14, 32]. 

### Whole health partners (veteran peers)

Peer support has been utilized for diverse conditions including mental illness, substance use, homelessness, diabetes, and cancer, with some positive outcomes noted, although more high-quality studies are needed [33–36]. In the Whole Health System, Whole Health Partners are Veterans who support fellow Veterans as they reflect on their health and well-being and explore what really matters in their lives. Not all Veterans engage regularly with their clinical care teams for a variety of reasons. Peer support offers a different way for Veterans to engage with VHA, without requiring that Veterans first connect with clinical services, if these are not needed.

### Patients

Collaborative practice includes both clinical and non-clinical health-related work with patients, families, and communities [4]. Interprofessional care typically does not include the patient as a “professional”, since a profession is defined as an occupation or career that requires considerable training and specialized study [37]. Yet, this is a more limited (and perhaps clinician-centric) understanding of “professional”, as patients clearly bring expertise about themselves and their own lived experiences and values, as well as unique strengths and skills, which are critical components of effective care plans [38, 39]. Patient-centered care and positive patient experience are associated with a range of improved clinical outcomes [40]. Federally standardized patient surveys to improve patient experience are employed widely across US health care, with mandatory reporting of survey results to consumers, acknowledging the importance of this information [41]. Explicitly elevating the patient to a full member of the interprofessional team with acknowledgment of their unique “professional” expertise in themselves may facilitate greater patient-centeredness of care and support a true transdisciplinary approach.

In the Whole Health System, conventional clinical care team members work seamlessly with these important new team members. Although an increase in number of team members poses the risk of even more fragmented care, the Whole Health approach provides a uniquely powerful antidote by elevating the Veteran as a full partner in their own health and well-being and placing what matters most to them explicitly at the center of care for all team members.

## The Whole Health approach and its impact

The Whole Health approach is an approach to care that focuses on the whole person, explicitly addressing the many diverse factors that can affect someone’s health and well-being, including the areas illustrated in the Circle of Health and Well-Being (Fig. 2), as well as the social, structural, and systemic determinants of health in the Whole Health for All graphic (Fig. 3). The Whole Health approach also centers care around what really matters to the individual. To truly support the whole person in these myriad ways necessitates an interprofessional workforce collaborating with each other and the patient. This was the impetus for expanding the care team and developing the infrastructure of the Whole Health System.

This type of whole-person, person-centered care is particularly necessary for effective care of US Veterans, who suffer from multiple chronic conditions at rates higher than the general US population, with one-third of Veterans living with three or more conditions [42, 43]. In addition, Veterans experience chronic pain and attempt suicide at significantly higher rates than the general US population [44, 45]. They also are more likely to be negatively affected by social determinants of health due to higher rates of poverty, homelessness, belonging to racial minorities, and being transgender [46–51]. Rates of trauma are also higher among Veterans than the general population, including childhood trauma and adverse childhood experiences, which can have a profound effect on future health outcomes [52–54]. 

Despite these complex patient demographics, a large national demonstration project of 18 diverse VHA facilities to implement a Whole Health System of care has shown promising early results. Use of Whole Health has been associated with substantial decreases in opioid use for Veterans with chronic pain, improved use of evidence-based therapies for Veterans with mental health conditions, and improved patient-reported outcomes including patient experience, engagement, self-care, perceived stress, pain, and notably, life meaning and purpose [55–58]. Life meaning and purpose, or MAP as previously defined, has been associated with suicide risk mitigation and also increased longevity, but it has not traditionally been a therapeutic target for health care systems [59, 60]. Focusing on MAP across the system by providing infrastructure to meaningfully assist Veterans in defining this for themselves and encouraging health care team members to prioritize what matters most to the Veteran may be effective in improving life meaning and purpose as well as enhancing health outcomes.

Others outside of VHA have also been using a Whole Health approach to care, though not yet at the same large scale as VHA [61]. The Whole Health approach has potential for diverse populations beyond just US Veterans, including for individuals living with chronic disease and those who have experienced trauma. According to the World Health Organization (WHO), 74% of deaths globally are related to chronic diseases [29]. In the WHO World Mental Health Surveys, 70% of respondents indicated they had experienced trauma, and over 30% have been exposed to four or more traumatic events [62, 63]. The impact of trauma on health outcomes has been well-established [54, 64]. A Whole Health approach may have potential in helping address the multi-faceted health effects associated with some of the traumas facing people globally today: poverty, war, persecution, refugee status, food crises and natural disasters related to global warming, sexual and other interpersonal violence, and other health inequities, through its emphasis on engaging, empowering and equipping individuals to take charge of their health and well-being in a manner aligned with trauma-informed care principles. This includes emphasis on balancing power, aligning with what really matters to the patient, and caring for the whole person, including taking into account social, structural and systemic drivers of health that may be present [65]. 

Additionally, through drawing in team members like coaches and peer partners, leveraging patients and families as essential care team members, and incorporating communities into care plans, broader opportunities exist for interprofessional team support even in areas where conventional health care may be more difficult to access. Indeed, the US National Academies of Science, Engineering and Medicine (NASEM) recently released a report, *Achieving Whole Health: A New Approach for Veterans and the Nation*, advocating that all US health systems should adopt a Whole Health approach for the health of the country. According to the NASEM report, education and training is a foundational element for Whole Health system transformation [61]. 

## Interprofessional Whole Health education across VHA

VHA’s large-scale system transformation over the past twelve years has been led by expansive educational efforts. VHA is uniquely positioned to impact health care system change in the US through its educational efforts, as both the largest provider of health professions education and training and the largest integrated health care system in the US [6, 9]. The majority of health professions training programs have now adopted interprofessional education competencies as part of program-level accreditation standards, and over 90% of institutions require interprofessional education for some or all students. When actually surveyed, however, over 21% of academic programs participating in interprofessional education answered that no more than half of their students in 2022 had participated in some level of interprofessional education activity [66, 67]. Part of this gap may be due to the fact that many practicing clinicians completed their training prior to this emphasis on interprofessional collaboration. High-quality programmatic interprofessional education has been characterized by experts as including advanced clinical interprofessional education experiences [68]. In order to ensure these advanced clinical interprofessional education experiences for trainees then, practicing clinicians must first be able to demonstrate these interprofessional competencies, so that learners are not unintentionally taught to devalue these because a hidden curriculum exists. The addition of novel roles in the VHA workforce such as chiropractors, acupuncturists, mindfulness teachers, health and wellness coaches, peer Whole Health Partners, etc., has made the need for interprofessional education, for both trainees and practicing clinicians, concrete and urgent. As outlined by the Interprofessional Education Collaborative (IPEC), (1) values and ethics, (2) roles and responsibilities, (3) communication, and (4) teamwork are all core areas where discrete knowledge and skills can be cultivated [68]. 

Since 2013, the VHA Office of Patient Centered Care and Cultural Transformation (OPCC&CT) has been steadily and intentionally building an array of interprofessional Whole Health courses, with course objectives tied closely to the four domains of core competencies for interprofessional collaborative practice. Indeed, the specific competencies identified in each of these domains align very closely with the foundational principles and philosophy of a Whole Health approach. As an example, Whole Health education courses have course objectives that support all 5 IPEC competencies related to Roles and Responsibilities: “1)Include the full scope of knowledge, skills, and attitudes of team members to provide care that is person-centered, safe, cost-effective, timely, efficient, effective, and equitable; 2) Collaborate with others within and outside of the health system to improve health outcomes; 3) Incorporate complementary expertise to meet health needs including the determinants of health; 4) Differentiate each team member’s role, scope of practice, and responsibility in promoting health outcomes; 5) Practice cultural humility in interprofessional teamwork.” [68].

The interprofessional educational effort across VHA has focused on accomplishing the following main goals to support whole Health transformation:

### Educate existing team members across the enterprise, especially clinicians, about a fundamental paradigm shift in health care

Whole Health education encourages all members of the team to look beyond disease management and treatment to what really matters most to each Veteran and to align care with this, through providing knowledge and skills for incorporating non-pharmacologic approaches for health and well-being, supporting whole-person Veteran self-care and clinical care, and meaningfully integrating new team members into the system. This shift towards a Whole Health approach is fostered by discussion and skill-building around the core competencies for interprofessional collaborative practice.

### Train new team members and professions

With the addition of new professions and roles to support the Whole Health System, VHA’s OPCC&CT developed training to provide the workforce necessary to support these novel positions across VHA. As of 2023, over 3,250 health and wellness coaches and 450 Whole Health Partners have been trained, as well as over 200 Whole Health Mentors (another new role in the Whole Health System developed to provide ongoing mentorship and training for staff supporting the Whole Health Pathway at individual facilities). As noted above, several CIH services became part of the standard benefits package for Veterans in 2017. Trainings have been developed and administered by OPCC&CT’s Integrative Health Coordinating Center to support some of these CIH approaches [69]. 

### Utilize an iterative approach

An iterative approach to course development, as well as to individual learning, has been important. Content revisions have occurred regularly through continuous process improvement and evaluation. Fidelity to Whole Health core concepts and values has been balanced with a desire to be responsive to additional priorities and changing realities over time. Content is adjusted for specific contexts and target audiences. For instance, clinical courses were originally three days long, designed to be immersive transformational in-person educational experiences. With increasing constraints on clinician time due to clinical needs and staffing issues, and subsequent demands of social distancing with the Covid-19 pandemic, several clinical courses are now two-hour virtual synchronous experiences, though they still aim to be immersive and transformational. In the past decade, Whole Health education overall has undergone substantial evolution in course content and length, mode of delivery, faculty development efforts, alignment with national priorities, and integration of important topics such as social determinants of health and diversity, equity, and inclusion, all informed by diverse stakeholder input and regular internal evaluation data. Additionally, for clinicians especially, understanding of Whole Health appears to be iterative, with use of a Whole Health approach evolving and maturing over time. Participation in Whole Health education longitudinally reinforces prior knowledge and supports acquisition of additional skills to further advance a Whole Health approach.

### Scale and spread

Whole Health education initially started with a single course and a small, centralized team to develop, coordinate, deliver, and evaluate impact. To reach large numbers of both staff and Veterans across VHA, an intentional effort to decentralize Whole Health education delivery was implemented over time. Currently, over 40% of formal Whole Health courses are delivered at local and regional levels by local and regional educators. Strategic partnerships have also been cultivated to support incorporation of Whole Health educational content into diverse educational offerings across VHA. As Whole Health has increasingly risen as a national priority, additional implementation supports have become available, including greater leadership support, funding, and educator time. While Whole Health education had taken an intentional voluntary approach to participation for many years, a large national initiative is currently underway for the integration of Whole Health into Primary Care, as well as Mental Health and Suicide Prevention efforts, across VHA. This national partnership has been instrumental in widespread delivery of Whole Health education, with national Whole Health training mandates to support the goal of reaching all VHA Primary Care and Mental Health teams over three years [11]. Despite being mandated against a backdrop of national staffing shortages, evaluation for the Primary Care course has demonstrated it has been well-received and effective in improving attitudes and adoption of Whole Health behaviors at follow-up [70]. 

There are currently over 90 different Whole Health educational offerings available to VHA employees, including face-to-face, virtual synchronous, and asynchronous offerings. Additional resource materials for self-directed learning are also available [71–73]. In the past decade, employees have completed over 30,000 face-to-face or virtual synchronous trainings and over 55,000 asynchronous trainings, per data from VHA’s educational tracking system. Table 1 describes examples of a few key courses that are offered as part of Whole Health training across VHA for which published evaluation data exist. While limited, the published work related to the impact of VHA’s Whole Health educational efforts for clinicians, the Whole Health Pathway, and health and wellness coaches (representing the three parts of the Whole Health System) has been promising. Results of a much larger internal evaluation effort have shown that implementation of interprofessional Whole Health education across a large integrated health care system has been feasible and well-received by course participants. Additionally, positive changes from pre- and post-training, as well as at follow-up, have been seen for attitudes and behaviors related to adopting a Whole Health approach for a significant number of Whole Health courses.

**Table 1:** Examples of Key VHA Whole Health Interprofessional Educational Offerings and Impact

Name	Description	Interprofessional Participation	Annual Participants	Effectiveness
*Whole Health in Your Practice*	Explains the Whole Health approach and takes clinician participants through all elements of the Circle of Health with multiple opportunities for skill-building. 20 h. Original clinical course for Whole Health education which is still widely attended. Delivered in-person previously and now more commonly as a virtual synchronous offering.	Over 13 professions represented in evaluations across 15 facilities (*N* = 633). In order of representation: Nurses, physicians, social workers, nurse practitioners psychologists, registered dieticians, respiratory therapists/occupational therapists/kinesiotherapists, administrators, medical support assistants, chaplain, patient-centered care coordinator, physician assistant, dentist, peer support specialist and other [88]	675 (average over 9 years of course deliveries)	Surveys of 655 participants from 15 VHA facilities done at 2-month follow-up (65% response rate) demonstrated significant positive changes in self-efficacy to engage in Integrative Medicine (IM) strategies, preparedness to discuss non-pharmaceutical approaches to care, and greater engagement in IM behaviors during clinical encounters. [88] In another study related to development of observational rating scales for evaluating patient-centered communication within a whole health approach to care, 65 clinical encounters across 8 providers before and after participating in this training were analyzed. Significant differences in quality of whole health goal setting and plan development were detected in participants after they attended this training [89].
*Whole Health for Mental Well-Being*	To support VHA’s Suicide Prevention efforts, with a focus on multiple aspects of mental health and wellness and how complementary approaches, self-care, and mindful awareness can support healthy thought patterns, behaviors, emotional states, and vitality levels. 14 h. Delivered in-person previously and now more commonly as a virtual synchronous offering.	Over 13 professions represented in pilot study across 2 facilities (*N* = 132). In order of representation: social worker, psychologist, nurse, physician, vocational/recreational/ physical therapist, health and wellness coach, nurse practitioner, peer support specialist, clinical pharmacist, registered dietitian, health systems specialist, other. 15% were Veterans [90].	208 (average over 5 years of course deliveries)	Statistically significant, large changes toward improvement from pre-test to post-test, with sustainment at follow up, were noted for multiple measures in analysis of 100 course participants. Measures included attitudes such as openness to use of CIH and self-efficacy around using CIH for self-care and discussing the Circle of Health; and also behaviors, such as use of CIH for patient care, working with Veterans on 5 aspects of mental health, discussing the Circle of Health components, use of CIH for self-care, and use of self-care strategies[90].
*Whole Health for Primary Care*	Brief introduction on how to use Whole Health for busy primary care teams. Significant focus on health and well-being of Primary Care team members. 2 h. Supports a national mandate to integrate Whole Health into Primary Care across VHA. Delivered as a virtual synchronous offering.	Designed for VHA core Primary Care teams, which include primary care providers, Licensed Practical Nurses, Registered Nurses, Medical Support Assistants (administrative support), as well as extended Primary Care team members, including Primary Care Mental Health Integration Team members (psychologists and psychiatrists), Social Workers, Clinical Pharmacists, Nutritionists and health and wellness coaches [91]	5,360 (actual number from most recent annual data; second year of national mandate to train all Primary Care teams)	Pilot evaluation of *Whole Health in Primary Care* demonstrated the training was effective in improving confidence about as well as integration of a whole health approach into care. For 463 participants responding to both pre-course and 45-day follow up surveys (42% response rate), participation in this course was associated with statistically significant changes with large effect sizes across all metrics, including knowledge, skills and attitudes as well as self-reported behavior changes related to the use of Whole Health skills in practice at 45-day follow-up [70].
*Whole Health Coaching*	Highly experiential and practical course provides instruction and mentoring on effective communication and coaching skills. Graduates are eligible to become National Board-Certified Health and Wellness Coaches. 42–79 h. Delivered in-person previously and now as a virtual synchronous offering.	In a study sample across 8 facilities (*N* = 258), the most common professions were nurses, social workers, psychologists, dieticians, pharmacists, peer support specialists, medical assistants, physical therapists and occupational therapists [92]. More recently, the course has been limited to those who will be serving formally in the position of health and wellness coach due to workforce demands and course capacity limitations.	340 (average over 9 years of course deliveries)	Early evaluation of using a mixed-methods analysis demonstrated that Whole Health Coaching was associated with improvements in mental health, perceived health competence and stress (small effect sizes), and Veterans reported being highly satisfied with their coaching experience [93].
*Taking Charge of My Life and Health Facilitator Training*	This train-the-facilitator course teaches Veteran peer facilitators how to lead the *Taking Charge of My Life and Health* group, a 9-week peer-led group program with an established curriculum that leverages the power of peer support to improve patient engagement, empowerment, health, and well-being among Veterans through Whole Health concepts, tools, and strategies. 30 h. Delivered in-person previously and now as a virtual synchronous offering.	As 30% of Veterans Administration employees are Veterans, participants who are peer facilitators represent a diverse range of professional backgrounds, including peer support specialists, social workers, psychologists, nutritionists, physical therapists, nurses, nurse practitioners, physicians, etc [94].	237 (average over 8 years of course deliveries)	Evaluation data *for Taking Charge of My Life and Health* groups demonstrated significant increases in self-care attitudes and behaviors, patient motivation, meaning and purpose, mental health, perceived stress, goal progress, and goal-specific hope. Sustained improvements at 2-month follow-up were seen for patient motivation, perceived stress, goal-specific hope, and goal progress. Significant gains were also noted in health care empowerment and physical health from pretest to follow-up [95], [96]. Participants of Taking Charge of My Life and Health Facilitator Training reported high levels of training satisfaction, quality, and utility, and sustained improvements in knowledge of Whole Health, self-efficacy for group facilitation, and self-efficacy for using Whole Health concepts and tools [97].

Beyond educational outcomes, additional downstream outcomes have been indirectly supported by educational efforts. Whole Health education alone has certainly not been sufficient for successful implementation, and yet, it has played a critical role [69]. Complex factors have impacted implementation of a Whole Health System across VHA, including federal legislation mandating that VHA explore additional ways to support Veterans with chronic pain in response to the opioid epidemic in the US, and a VHA Directive mandating that CIH services be part of the standard benefits package for Veterans [11]. Combining such policies with implementation supports and foundational Whole Health education has supported implementation successes, including the increased use of Whole Health across VHA, the increase in staff involvement in Whole Health (with a correlation with greater resilience, improved stress management skills, and better management of workplace burnout), and the promising results discussed above on a variety of important care measures for Veterans from the early Whole Health System evaluation of 18 Whole Health Flagship sites across VHA [11, 55–58, 74, 75]. 

Additional impact may occur through exposure of a Whole Health approach to health professions trainees. VHA is the largest training provider of health professions education and training across the US, with over 120,000 trainees annually. Of practicing physicians across the US, 70% percent received at least some of their training within VHA [76]. Learning and professional identity formation occurring in this type of clinical training environment focused on collaborative practice in support of the whole person and what matters most to this person may have important downstream effects in terms of future attitudes towards collaborative practice. Stakeholders from medical and nursing schools, as well as residency and fellowship programs from several disciplines and sites across the US, have consulted with Whole Health education team members, as well as local Whole Health champions, about incorporating Whole Health into their curricula. Additionally, the spread of Whole Health education has impacted other groups, such as the US Army Surgeon General’s Office, which collaborated with VHA to adopt Whole Health principles and create *Move2Health*, the Army’s own version of VHA’s flagship Whole Health clinical course [77]. Across VHA, researchers have been learning about Whole Health and designing interventions and evaluation strategies, and funding has also been available to support these [78]. Additionally, VHA Whole Health educational materials developed with federal funds are considered taxpayer property and therefore widely available for use. For example, VHA’s Whole Health website offers a large array of online resources available for free to all [71–73]. Such education and research connections have resulted in an increase in Whole Health publications, workshops, and presentations both inside and outside of VHA in recent years. A PubMed search using the search term “Whole Health” yielded 13 total results in 2014 and now yields nearly 100 publications related to Whole Health efforts within VHA and the US Department of Defense.

## Lessons learned

Health systems and professional education programs have increasingly prioritized interprofessional care, but its full potential remains unrealized. Many possible reasons exist, including limitations related to knowledge, skills and resources that exist at individual, team, and organizational levels [79, 80]. Effective interprofessional education uses different approaches in facing unique challenges [81]. The past decade’s experience with implementing Whole Health education in support of system transformation across VHA has provided many learning opportunities. Three key lessons may help others who are working to provide interprofessional education and collaborative care across an entire system:

### Nurture an expansive and inclusive view of collaborative care through transformative educational and strengths-based approaches

The Whole Health System, which incorporates new team members who explicitly support health and well-being and includes patients as full partners, is a model promoting shared responsibility for health, co-creation of health plans, and an expanded understanding of the term “expertise” in the health care system. Inherent in this change is a greater leveling of power across the health care system, which may cause tension for those who have already been working in the current model. Whole Health education uses a transformative educational approach, with core elements of critical reflection, dialogue, individual experience, and context [82, 83]. Participants face questions during the courses such as:

- Is the current health care system working?
- Are we focusing on what really matters?
- Is my own current work effective?
- What might it be like to have a more expansive and inclusive team?

Participants are invited to reflect on these questions with mindful awareness, which is foundational to the Whole Health approach. In this place of mindful self-reflection, participants are engaged in a dilemma with the current health care paradigm, and often become motivated to resolve the tension of where they are and where they want to be, through changes in their thoughts and actions. To be effective across a large diverse health care system, this type of approach has required purposeful attention to the range of participants and perspectives across the system, with course content and activities intentionally designed to meet each person where they are on their Whole Health journey. Space is provided for personal reflection, with open discussion of barriers and concerns in a safe and nonjudgmental environment. Time is dedicated to group brainstorming of potential solutions to problems.

Another key factor in the success of Whole Health interprofessional education has been using a strengths-based approach. Celebrating strengths and successes of patients is a fundamental component of a Whole Health approach and is explicitly taught in courses, often with accompanying skill-building activities. This approach often is modeled in courses by faculty. Faculty are intentionally interprofessional (except for a few more specialized courses), so that throughout the course, participants experience faculty modeling trust and collaboration between different professions. Additionally, many professional disciplines (social workers, psychologists, etc.) come to Whole Health educational offerings already closely aligned with a Whole Health approach, with significant experience using a whole-person, strengths-based and values-based approach to care. Inviting these participants to share their expertise during courses provides a powerful educational opportunity for other participants and honors important work these professions have been doing. This has been important strategically with historical programs and efforts, also, so that VHA programs and teams do not feel that Whole Health is a program seeking to devalue or displace their historical and current work; rather, it is seen as a welcome resource and partnership that aligns with, supports, and expands their own work and encourages others to buy in.

A strengths-based approach is also used with respect to course target audiences. While courses may have certain target audiences (such as clinicians), participants from different interprofessional backgrounds outside of the intended target audience often attend, due to their interest in the different course content. While one administrative approach would be to strictly limit participation by professional background, making course design and facilitation less complicated, this is not the approach Whole Health education has generally taken. With a wide variety of professional backgrounds in nearly all courses, faculty intentionally create an inclusive environment for learners, highlighting the unique perspectives and strengths of different types of participants, while simultaneously being mindful of scope of practice issues. Using this inclusive, strengths-based approach, a Whole Health course may allow a primary care physician, for example, not only to learn about new roles in VHA, but also to interact with and appreciate diverse perspectives and expertise from colleagues such as a health and wellness coach, a Whole Health Partner, or an acupuncturist, not to mention Veterans who are VHA patients themselves, given that 30% of Veterans Administration employees are Veterans [94]. The result is a richer interprofessional experience that also fosters team-building and networking, in support of future interprofessional collaboration.

### Balance aspiration with acknowledgment of differing resources and realities

The Whole Health model engages multiple team members across the entire health care system to support whole person, person-centered care that equips and empowers people to take charge of their own lives. In this respect, it may feel remarkably aspirational, especially to an under-resourced, over-tasked clinician working in a system largely focused on disease and disability. Clinicians may be less interested in additional novel team members if they are feeling overwhelmed by the lack of more basic team members, such as when primary care clinics or inpatient wards do not have enough nurses or administrative clerks to optimally meet Veterans’ needs. Genuine acknowledgment of these realities can facilitate interprofessional care being viewed as a potential solution to current challenges and a means for improving people’s experiences in a health care system, rather than something that discounts a current distressing reality.

Whole Health System implementation across VHA currently differs from one facility to the next. Discussions during a course about the aspirational state may result in participants becoming aware of discrepancies from one site to another, such as variance in the availability of team members (e.g., CIH practitioners, and health and wellness coaches) among locations. Early evaluation work for participants attending the initial multi-day clinical course actually indicated a possible increase in burnout at 60-day follow up. One hypothesis for this finding was that participants became excited about the possibilities of Whole Health during the course, but upon returning to their regular clinical work and attempting to integrate what they learned without appropriate supports for effective practice change, they became more fatigued and discouraged than before they had learned about Whole Health. Just as behavior change with patients is more effective with realistic goals, action planning, and preparation, an important course strategy that has evolved over time includes having participants develop their own practical and realistic goals for incorporating Whole Health, based on the resources available to them. The presence of leadership support, in particular, has been an important factor in terms of more successful post-course implementation. Whole Health has now been identified as one of VHA’s top national priorities [84]; for individual facilities and teams, however, differing levels of leadership support for Whole Health may exist due to multiple competing local priorities and contexts. Understanding the degree of leadership support for Whole Health transformation and the expectations of local leadership after attending Whole Health courses has been important for participants.

### Co-create a positive interprofessional experience using a person-centered approach and relational skill-building

As noted above, courses provide an interprofessional educational experience that not only tells, but actually shows, participants how to work interprofessionally through welcoming people with different backgrounds, embracing a strengths-based and inclusive approach that honors diverse professions and expertise and emphasizes peer learning, or “learning from the wisdom of the group.” During courses, participants are invited to reflect on what really matters to them, in their personal lives, providing an opportunity for participants to understand human commonalities across diverse backgrounds, and to feel “re-humanized” in the health care system. Intentional reflection and discussion about what really matters professionally spotlights common goals and sense of purpose shared by all team members, which is critical for high-functioning teams [85]. 

Courses focus significantly on relational, versus transactional, communication and interactions, ranging from learning how to ask someone what really matters to skill-building around generous listening, empathy, shared goal-setting, and collaboration. Experiences in mindfulness are nearly universal across all courses, providing an opportunity for skill-building focused on bringing kind curiosity to the present moment. Discussions focused on social, structural and systemic drivers of health include how unconscious bias may show up in interactions between human beings. These types of diverse skills are helpful with patient interactions and with interprofessional team dynamics [86, 87]. Synchronous courses also include opportunities for mindful movement, laughter, and sharing of experiences; this furthers human connection and a sense of VHA staff being not just workers, but whole persons themselves. As a result, participants learn from direct experience, rather than intellectualizing, how being in an interprofessional environment is a positive experience. They see firsthand how they can collaborate to co-create Whole Health initiatives and effective interprofessional care together.

## Conclusion

In the past decade, Whole Health interprofessional education across VHA has taught staff and Veterans about a new model of collaborative practice, the Whole Health System, supporting development and integration of key new interprofessional team members, and spreading a new paradigm of care that focuses on the whole person and asks Veterans not, “What’s the matter with you?” but rather, “What matters to you?” Educational programming has also provided Veterans, as the most important team members, with enhanced skills for exploring and supporting their own health and well-being, in alignment with their MAP. A decade ago, only a small handful of employees and Veterans across VHA had heard the term “Whole Health”. Now, Whole Health is one of VHA’s top national priorities [84]. Interprofessional education and many other facilitators have culminated in high levels of support and increased implementation of Whole Health System transformation across VHA.

What has been learned in the past decade of large-scale interprofessional Whole Health education can guide other organizations as they explore how they, too, can further support interprofessional collaborative practice to promote person-centered, whole person health. Key lessons from VHA’s first decade of education to support Whole Health transformation include the power of transformative education that focuses on strengths, the importance of balancing aspiration with realities, and the impact of facilitating interprofessional groups to come together to co-create positive experiences that demonstrate both how to share power across diverse and new roles and how to bring awareness back to the shared humanity that can exist in health care. Importantly, VHA has successfully demonstrated that such an ambitious educational effort can be scaled and spread across a large health care organization. Furthermore, a large number of VHA Whole Health educational materials are widely available to the public, to support other organizations wishing to support interprofessional collaborative practice in support of whole person health and well-being [71–73]. 

As practicing clinicians and clinician-educators within VHA continue to practice collaboratively using a Whole Health approach, vast numbers of current and future health professions trainees will have clinical training experiences that include collaborative practice as part of the hidden curriculum, with the potential to further shift US health care in general. Beyond the US, a Whole Health approach has tremendous potential for people everywhere, especially with growing prevalence of chronic disease worldwide, through empowering and equipping people, balancing power across care teams, centering patient voice and choice, caring for the whole person, and expanding care team expertise. This has the potential to provide the most inclusive, culturally-responsive, trauma-informed care and healing available, both for any given individual and, on a broader scale, across communities.

## Data Availability

No datasets were generated or analysed during the current study.

## Abbreviations

CIH: Complementary and Integrative Health
IPEC: Interprofessional Education Collaborative
MAP: Mission, Aspiration and/or Purpose
NASEM: National Academies of Sciences Engineering and Medicine
OPCC&CT: Office of Patient Centered Care & Cultural Transformation
US: United States
VHA: Veterans Health Administration
WHO: World Health Organization

## Declarations

### Purpose statement

Whole Health is an approach to health care that empowers and equips people to take charge of their health and well-being and to live their lives to the fullest. In 2011, the US Veterans Health Administration (VHA) created the Office of Patient Centered Care & Cultural Transformation to implement a Whole Health approach and change how health care is delivered to Veterans across the enterprise. This health care transformation has included shifting to more of a focus on whole person care for health and well-being that aligns with what matters most to each patient, as well as the formal incorporation of health care professionals who are novel to mainstream health care systems. Such cultural change has necessitated interprofessional education for practicing clinicians who previously trained in a different health care paradigm. This commentary describes the interprofessional education initiatives and lessons learned from the first decade of health care transformation towards a Whole Health approach across VHA, and demonstrates the critical role of ongoing interprofessional education for health systems to effectively promote collaborative practice for whole person health and well-being. This approach to care can potentially benefit additional populations and other health care systems as well, and VHA’s experience offers insights for health professions educators regarding novel ways that interprofessional teams can be developed to transform health care.
